# Propane-1,3-diaminium bis­(tetra­fluoro­borate)–18-crown-6 (1/2)

**DOI:** 10.1107/S1600536812001572

**Published:** 2012-01-18

**Authors:** Min-Min Zhao

**Affiliations:** a Ordered Matter Science Research Center, College of Chemistry and Chemical Engineering, Southeast University, Nanjing 210096, People’s Republic of China

## Abstract

In the title compound, C_3_H_12_N_2_^2+^·2BF_4_^−^·2C_12_H_24_O_6_, the central C atom of the propane-1,3-diammonium cation lies on a crystallographic twofold rotation axis. The terminal NH_3_^+^ groups insert into the crown rings through strong N—H⋯O hydrogen-bonding inter­actions, resulting in the formation of a 1:2 supra­molecular [(C_3_H_12_N_2_)·(C_12_H_24_O_6_)_2_]^2+^ complex. The anions are linked to the supra­molecular complexes *via* weak C—H⋯F hydrogen bonds. The F atoms of the anion are disordered over two orientations with site occupancies of 0.5.

## Related literature

For the structures and properties of a related compounds, see: Fu *et al.* (2011 ▶); Zhao (2012 ▶) and references therein.
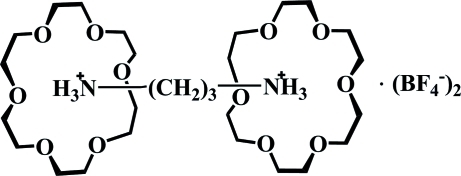


## Experimental

### 

#### Crystal data

C_3_H_12_N_2_^2+^·2BF_4_^−^·2C_12_H_24_O_6_
*M* *_r_* = 778.39
Monoclinic, 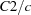

*a* = 22.615 (5) Å
*b* = 8.8423 (18) Å
*c* = 21.077 (4) Å
β = 113.41 (3)°
*V* = 3867.8 (16) Å^3^
*Z* = 4
Mo *K*α radiation
μ = 0.12 mm^−1^
*T* = 298 K
0.10 × 0.05 × 0.05 mm

#### Data collection

Rigaku Mercury2 diffractometer
Absorption correction: multi-scan (*CrystalClear*; Rigaku, 2005 ▶) *T*_min_ = 0.910, *T*_max_ = 1.000
16100 measured reflections
3413 independent reflections
2018 reflections with *I* > 2σ(*I*)
*R*_int_ = 0.079

#### Refinement

*R*[*F* ^2^ > 2σ(*F* ^2^)] = 0.077
*wR*(*F* ^2^) = 0.212
*S* = 1.13
3413 reflections
268 parameters
37 restraints
H-atom parameters constrained
Δρ_max_ = 0.31 e Å^−3^
Δρ_min_ = −0.23 e Å^−3^

### 

Data collection: *CrystalClear* (Rigaku, 2005 ▶); cell refinement: *CrystalClear*; data reduction: *CrystalClear*; program(s) used to solve structure: *SHELXS97* (Sheldrick, 2008 ▶); program(s) used to refine structure: *SHELXL97* (Sheldrick, 2008 ▶); molecular graphics: *SHELXTL* (Sheldrick, 2008 ▶); software used to prepare material for publication: *SHELXTL*.

## Supplementary Material

Crystal structure: contains datablock(s) I, global. DOI: 10.1107/S1600536812001572/rz2697sup1.cif

Structure factors: contains datablock(s) I. DOI: 10.1107/S1600536812001572/rz2697Isup2.hkl

Additional supplementary materials:  crystallographic information; 3D view; checkCIF report

## Figures and Tables

**Table 1:** Hydrogen-bond geometry (Å, °)

*D*—H⋯*A*	*D*—H	H⋯*A*	*D*⋯*A*	*D*—H⋯*A*
N1—H1*B*⋯O2	0.89	2.06	2.915 (3)	161
N1—H1*A*⋯O4	0.89	2.03	2.911 (4)	169
N1—H1*C*⋯O6	0.89	2.08	2.967 (4)	179
C12—H12*B*⋯F4′	0.97	2.48	3.316 (19)	144
C13—H13*A*⋯F2	0.97	2.47	3.346 (12)	150
C13—H13*A*⋯F2′	0.97	2.42	3.361 (16)	162
C5—H5*A*⋯F3′^i^	0.97	2.41	3.350 (18)	162
C10—H10*B*⋯F1′^ii^	0.97	2.41	3.355 (17)	166
C10—H10*B*⋯F2′^ii^	0.97	2.44	3.235 (19)	139
C8—H8*A*⋯F3′^iii^	0.97	2.50	3.412 (14)	156

Symmetry codes: (i) 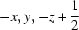; (ii) 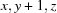; (iii) 
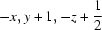
.

**Fig. 1.**
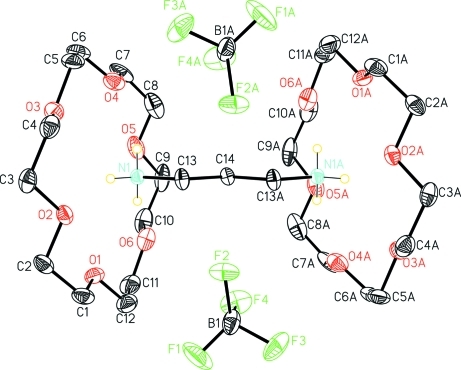
The structure of the title compound with displacement ellipsoids drawn at the 30% probability level. Only H atoms of the NH3+ groups are shown. Symmetry code: (A) -x, +y, 1/2-z.

**Fig. 2.**
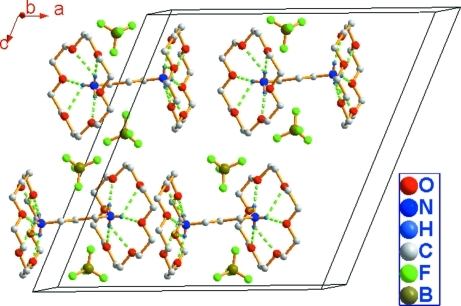
Crystal packing of the title compound approximately viewed along the b axis. Only hydrogen atoms involved in N—H···O hydrogen bonds (dashed line) are shown.

## supplementary crystallographic information

### Comment

As a continuation of the research project devoted to the synthesis and characterization of novel phase transition crystals of amino compounds (Zhao, 2012), the crystal structure of the title compound is reported herein.

The title compound is composed of [(C_3_H_12_N_2_).(C_12_H_24_O_6_)_2_]^2+^ cations and BF_4_^-^ anions (Fig. 1). A 1:2 supramolecular rotator-stator structure is generated between one propane-1,3-diammonium dication and two 18-crown-6 molecules through six N—H···O hydrogen bonds (Table 1) occurring between the protons of the NH_3_^+ ^ groups and the O atoms of the crown ethers. The supramolecular rotator has crystallographically imposed twofold rotation symmetry, the central C atom of the propane-1,3-diammonium cation lying on a crystallographic twofold rotation axis. The macrocycle adopts a conformation with approximate D_3d_ symmetry, with all O-C-C-O torsion angles being *gauche* and alternating in sign, and all C-O-C-C torsion angles being *trans*. The C–N bonds of the cation are almost perpendicular to the mean planes of the oxygen atoms of the crown ethers. The supramolecular structure is introduced as counter cation to BF_4_^-^ anions. The B atom has a flattened tetrahedral coordination geometry provided by four F atoms [range of *cis*-bond angles = 121.6 (5)-89.1 (9) °; dav(F-B) = 1.226 (9)-1.445 (9)Å]. All F atoms of the BF_4_^-^ anion are disordered over two orientations. In the crystal structure (Fig. 2), cations and anions are linked by weak interionic C—H···F hydrogen bonds (Table 1).

### Experimental

Commercial 18-crown-6 (6 mmol), HBF_4_ (6 mmol) and propane-1,3-diamine (3 mmol) were dissolved in a water/EtOH (1:1 *v*/*v*) solution. The solvent was slowly evaporated in air affording colourless block-shaped crystals of the title compound suitable for X-ray analysis.

The dielectric constant of the title compound as a function of temperature indicates that the permittivity is basically temperature-independent, suggesting that this compound should be not a real ferroelectrics or there may be no distinct phase transition occurred within the measured temperature range. Similarly, below the melting point (412 K) of the compound, the dielectric constant as a function of temperature also goes smoothly, and there is no dielectric anomaly observed (dielectric constant ranging from 4.5 to 8.8).

### Refinement

All H atoms attached to C atoms were fixed geometrically and treated as riding with 0.97 Å and *U_iso_*(H) = 1.2*U_eq_*(C). The positional parameters of the N-bound H atoms were intially refined freely, subsequently they were restrained using a N–H distance of 0.89 (2) Å, and in the final refinements treated as riding with *U_iso_*(H) = 1.5*U_eq_*(N). All F atoms were disordered over two sites with occupancies of 0.5, and were refined anisotropically using ADP restraints (SIMU and DELU).

### Crystal data

**Table:**

C_3_H_12_N_2_^2+^·2BF_4_^−^·2C_12_H_24_O_6_	*F*(000) = 1656
*M**_r_* = 778.39	*D*_x_ = 1.337 Mg m^−^^3^
Monoclinic, *C*2/*c*	Mo *K*α radiation, λ = 0.71073 Å
Hall symbol: -C 2yc	Cell parameters from 3410 reflections
*a* = 22.615 (5) Å	θ = 2.9–27.5°
*b* = 8.8423 (18) Å	µ = 0.12 mm^−^^1^
*c* = 21.077 (4) Å	*T* = 298 K
β = 113.41 (3)°	Block, colourless
*V* = 3867.8 (16) Å^3^	0.10 × 0.05 × 0.05 mm
*Z* = 4	

### Data collection

**Table:**

Rigaku Mercury2 diffractometer	3413 independent reflections
Radiation source: fine-focus sealed tube	2018 reflections with *I* > 2σ(*I*)
graphite	*R*_int_ = 0.079
Detector resolution: 13.6612 pixels mm^-1^	θ_max_ = 25.0°, θ_min_ = 3.0°
CCD profile fitting scans	*h* = −26→26
Absorption correction: multi-scan (*CrystalClear*; Rigaku, 2005)	*k* = −10→10
*T*_min_ = 0.910, *T*_max_ = 1.000	*l* = −25→25
16100 measured reflections	

### Refinement

**Table:**

Refinement on *F*^2^	Primary atom site location: structure-invariant direct methods
Least-squares matrix: full	Secondary atom site location: difference Fourier map
*R*[*F*^2^ > 2σ(*F*^2^)] = 0.077	Hydrogen site location: inferred from neighbouring sites
*wR*(*F*^2^) = 0.212	H-atom parameters constrained
*S* = 1.13	*w* = 1/[σ^2^(*F*_o_^2^) + (0.080*P*)^2^ + 3.2068*P*] where *P* = (*F*_o_^2^ + 2*F*_c_^2^)/3
3413 reflections	(Δ/σ)_max_ < 0.001
268 parameters	Δρ_max_ = 0.31 e Å^−^^3^
37 restraints	Δρ_min_ = −0.22 e Å^−^^3^

### Special details

**Table:**

Geometry. All esds (except the esd in the dihedral angle between two l.s. planes) are estimated using the full covariance matrix. The cell esds are taken into account individually in the estimation of esds in distances, angles and torsion angles; correlations between esds in cell parameters are only used when they are defined by crystal symmetry. An approximate (isotropic) treatment of cell esds is used for estimating esds involving l.s. planes.
Refinement. Refinement of F^2^ against ALL reflections. The weighted R-factor wR and goodness of fit S are based on F^2^, conventional R-factors R are based on F, with F set to zero for negative F^2^. The threshold expression of F^2^ > 2sigma(F^2^) is used only for calculating R-factors(gt) etc. and is not relevant to the choice of reflections for refinement. R-factors based on F^2^ are statistically about twice as large as those based on F, and R- factors based on ALL data will be even larger.

### Fractional atomic coordinates and isotropic or equivalent isotropic displacement parameters (Å^2^)

**Table:**

	*x*	*y*	*z*	*U*_iso_*/*U*_eq_	Occ. (<1)
O2	0.19727 (10)	0.6715 (3)	0.26770 (12)	0.0558 (6)	
O3	0.15021 (11)	0.7740 (3)	0.12991 (13)	0.0650 (7)	
N1	0.10547 (11)	0.9201 (3)	0.23703 (13)	0.0433 (7)	
H1A	0.0918	0.9641	0.1957	0.065*	
H1B	0.1379	0.8578	0.2419	0.065*	
H1C	0.1189	0.9907	0.2698	0.065*	
O1	0.22029 (12)	0.8928 (3)	0.37179 (12)	0.0646 (7)	
O4	0.07376 (13)	1.0406 (3)	0.09880 (15)	0.0786 (9)	
O5	0.10078 (13)	1.2567 (3)	0.20532 (18)	0.0850 (9)	
O6	0.14972 (14)	1.1589 (3)	0.34468 (17)	0.0771 (8)	
C2	0.25128 (17)	0.6683 (4)	0.3313 (2)	0.0652 (11)	
H2A	0.2862	0.7248	0.3269	0.078*	
H2B	0.2654	0.5647	0.3431	0.078*	
C3	0.21010 (19)	0.5983 (4)	0.2141 (2)	0.0652 (11)	
H3A	0.2180	0.4915	0.2244	0.078*	
H3B	0.2483	0.6418	0.2112	0.078*	
C6	0.0933 (2)	0.9686 (6)	0.0513 (2)	0.0876 (15)	
H6A	0.1345	1.0089	0.0555	0.105*	
H6B	0.0621	0.9881	0.0047	0.105*	
C1	0.2343 (2)	0.7359 (5)	0.3861 (2)	0.0714 (12)	
H1D	0.1971	0.6845	0.3879	0.086*	
H1E	0.2700	0.7243	0.4306	0.086*	
C7	0.0639 (2)	1.2022 (6)	0.0872 (3)	0.0953 (18)	
H7A	0.1024	1.2499	0.0868	0.114*	
H7B	0.0289	1.2203	0.0427	0.114*	
C5	0.0984 (2)	0.8034 (5)	0.06412 (19)	0.0801 (13)	
H5A	0.0580	0.7645	0.0633	0.096*	
H5B	0.1075	0.7525	0.0282	0.096*	
C10	0.1474 (3)	1.3118 (5)	0.3255 (3)	0.0974 (16)	
H10A	0.1872	1.3399	0.3215	0.117*	
H10B	0.1429	1.3738	0.3613	0.117*	
C4	0.1550 (2)	0.6196 (5)	0.1473 (2)	0.0736 (12)	
H4A	0.1616	0.5609	0.1118	0.088*	
H4B	0.1157	0.5848	0.1509	0.088*	
C11	0.2008 (3)	1.1288 (6)	0.4107 (2)	0.0910 (15)	
H11A	0.1929	1.1804	0.4472	0.109*	
H11B	0.2412	1.1654	0.4106	0.109*	
C9	0.0925 (3)	1.3385 (5)	0.2604 (3)	0.0963 (17)	
H9A	0.0528	1.3078	0.2641	0.116*	
H9B	0.0895	1.4458	0.2499	0.116*	
C8	0.0481 (2)	1.2698 (6)	0.1433 (4)	0.1030 (18)	
H8A	0.0369	1.3756	0.1333	0.124*	
H8B	0.0114	1.2179	0.1462	0.124*	
C12	0.2061 (2)	0.9645 (6)	0.4233 (2)	0.0851 (13)	
H12A	0.2395	0.9425	0.4684	0.102*	
H12B	0.1654	0.9272	0.4224	0.102*	
B1	0.0793 (3)	0.6074 (7)	0.4303 (3)	0.0773 (15)	
C14	0.0000	0.9285 (5)	0.2500	0.0449 (11)	
H14A	0.0184	0.9930	0.2904	0.054*	
C13	0.05211 (15)	0.8336 (4)	0.2431 (2)	0.0552 (9)	
H13A	0.0699	0.7687	0.2834	0.066*	
H13B	0.0330	0.7690	0.2028	0.066*	
F2	0.0550 (6)	0.5710 (10)	0.3586 (6)	0.090 (3)	0.50
F3	0.0439 (7)	0.5494 (15)	0.4626 (7)	0.110 (5)	0.50
F4	0.0679 (5)	0.7681 (8)	0.4203 (4)	0.118 (3)	0.50
F1	0.1357 (3)	0.5838 (17)	0.4687 (6)	0.163 (5)	0.50
F1'	0.1185 (8)	0.4723 (15)	0.4546 (6)	0.220 (6)	0.50
F2'	0.0789 (8)	0.5857 (18)	0.3725 (7)	0.172 (7)	0.50
F3'	0.0256 (7)	0.5942 (19)	0.4373 (9)	0.166 (7)	0.50
F4'	0.1152 (8)	0.7196 (18)	0.4632 (7)	0.186 (6)	0.50

### Atomic displacement parameters (Å^2^)

**Table:**

	*U*^11^	*U*^22^	*U*^33^	*U*^12^	*U*^13^	*U*^23^
O2	0.0451 (13)	0.0560 (14)	0.0655 (16)	0.0094 (11)	0.0213 (12)	0.0071 (12)
O3	0.0583 (15)	0.0699 (17)	0.0626 (17)	−0.0108 (13)	0.0197 (13)	−0.0076 (13)
N1	0.0392 (14)	0.0452 (15)	0.0476 (15)	0.0051 (12)	0.0196 (12)	0.0064 (12)
O1	0.0721 (17)	0.0758 (17)	0.0456 (14)	−0.0101 (14)	0.0230 (13)	0.0011 (12)
O4	0.0700 (18)	0.088 (2)	0.0635 (18)	−0.0096 (15)	0.0110 (14)	0.0276 (16)
O5	0.0607 (18)	0.0645 (18)	0.128 (3)	0.0193 (14)	0.0355 (18)	0.0222 (17)
O6	0.093 (2)	0.0527 (16)	0.104 (2)	−0.0128 (15)	0.0591 (19)	−0.0153 (15)
C2	0.048 (2)	0.066 (2)	0.071 (3)	0.0021 (18)	0.011 (2)	0.024 (2)
C3	0.074 (3)	0.045 (2)	0.092 (3)	0.0139 (19)	0.049 (2)	0.0021 (19)
C6	0.069 (3)	0.125 (4)	0.045 (2)	−0.035 (3)	−0.002 (2)	0.018 (3)
C1	0.068 (3)	0.074 (3)	0.057 (2)	−0.004 (2)	0.009 (2)	0.027 (2)
C7	0.059 (3)	0.097 (4)	0.088 (3)	−0.019 (2)	−0.015 (2)	0.061 (3)
C5	0.069 (3)	0.112 (4)	0.044 (2)	−0.026 (2)	0.006 (2)	−0.014 (2)
C10	0.113 (4)	0.065 (3)	0.144 (5)	−0.001 (3)	0.083 (4)	−0.027 (3)
C4	0.078 (3)	0.072 (3)	0.081 (3)	−0.009 (2)	0.043 (3)	−0.023 (2)
C11	0.099 (3)	0.111 (4)	0.069 (3)	−0.025 (3)	0.040 (3)	−0.037 (3)
C9	0.100 (4)	0.046 (2)	0.183 (6)	0.021 (3)	0.100 (4)	0.011 (3)
C8	0.066 (3)	0.071 (3)	0.160 (6)	0.007 (2)	0.032 (4)	0.047 (3)
C12	0.087 (3)	0.108 (4)	0.062 (3)	−0.019 (3)	0.032 (2)	−0.012 (3)
B1	0.073 (4)	0.090 (4)	0.087 (4)	−0.020 (3)	0.051 (3)	−0.033 (3)
C14	0.038 (2)	0.038 (2)	0.059 (3)	0.000	0.020 (2)	0.000
C13	0.0443 (18)	0.0430 (18)	0.084 (3)	−0.0031 (16)	0.0320 (18)	−0.0038 (18)
F2	0.118 (7)	0.076 (4)	0.053 (4)	0.018 (4)	0.010 (4)	0.005 (3)
F3	0.155 (12)	0.100 (6)	0.111 (6)	−0.061 (7)	0.091 (7)	−0.012 (4)
F4	0.178 (8)	0.075 (4)	0.099 (5)	−0.020 (4)	0.052 (5)	−0.016 (3)
F1	0.057 (4)	0.207 (10)	0.160 (8)	−0.022 (5)	−0.025 (4)	0.059 (9)
F1'	0.347 (17)	0.195 (9)	0.148 (9)	0.138 (11)	0.131 (10)	0.066 (8)
F2'	0.168 (14)	0.252 (14)	0.091 (8)	0.078 (9)	0.048 (9)	−0.038 (7)
F3'	0.094 (7)	0.203 (15)	0.246 (18)	−0.048 (8)	0.115 (11)	−0.116 (11)
F4'	0.227 (13)	0.193 (9)	0.198 (12)	−0.162 (10)	0.146 (10)	−0.128 (10)

### Geometric parameters (Å, °)

**Table:**

O2—C2	1.410 (4)	C10—C9	1.459 (7)
O2—C3	1.429 (4)	C10—H10A	0.9700
O3—C4	1.407 (5)	C10—H10B	0.9700
O3—C5	1.439 (4)	C4—H4A	0.9698
N1—C13	1.477 (4)	C4—H4B	0.9701
N1—H1A	0.8900	C11—C12	1.473 (7)
N1—H1B	0.8900	C11—H11A	0.9699
N1—H1C	0.8900	C11—H11B	0.9699
O1—C12	1.400 (5)	C9—H9A	0.9699
O1—C1	1.428 (5)	C9—H9B	0.9700
O4—C6	1.397 (6)	C8—H8A	0.9700
O4—C7	1.452 (5)	C8—H8B	0.9700
O5—C8	1.380 (6)	C12—H12A	0.9699
O5—C9	1.442 (6)	C12—H12B	0.9700
O6—C10	1.405 (5)	B1—F1	1.226 (9)
O6—C11	1.436 (6)	B1—F2'	1.228 (15)
C2—C1	1.481 (6)	B1—F3'	1.286 (14)
C2—H2A	0.9701	B1—F4'	1.296 (9)
C2—H2B	0.9700	B1—F3	1.342 (11)
C3—C4	1.475 (6)	B1—F2	1.423 (13)
C3—H3A	0.9699	B1—F4	1.445 (9)
C3—H3B	0.9699	B1—F1'	1.454 (11)
C6—C5	1.482 (6)	C14—C13	1.501 (4)
C6—H6A	0.9701	C14—C13^i^	1.501 (4)
C6—H6B	0.9700	C14—H14A	0.9700
C1—H1D	0.9701	C13—H13A	0.9701
C1—H1E	0.9699	C13—H13B	0.9700
C7—C8	1.490 (7)	F2—F2'	0.52 (3)
C7—H7A	0.9700	F3—F3'	0.66 (2)
C7—H7B	0.9699	F4—F4'	1.174 (18)
C5—H5A	0.9700	F1—F1'	1.058 (15)
C5—H5B	0.9700	F1—F4'	1.275 (16)
			
C2—O2—C3	111.8 (3)	O5—C9—C10	110.0 (4)
C4—O3—C5	112.0 (3)	O5—C9—H9A	110.2
C13—N1—H1A	109.5	C10—C9—H9A	110.3
C13—N1—H1B	109.5	O5—C9—H9B	109.1
H1A—N1—H1B	109.5	C10—C9—H9B	109.2
C13—N1—H1C	109.5	H9A—C9—H9B	108.0
H1A—N1—H1C	109.5	O5—C8—C7	109.2 (4)
H1B—N1—H1C	109.5	O5—C8—H8A	109.8
C12—O1—C1	112.1 (3)	C7—C8—H8A	109.7
C6—O4—C7	113.5 (4)	O5—C8—H8B	109.8
C8—O5—C9	112.4 (4)	C7—C8—H8B	109.9
C10—O6—C11	112.4 (4)	H8A—C8—H8B	108.4
O2—C2—C1	109.8 (3)	O1—C12—C11	109.5 (4)
O2—C2—H2A	109.3	O1—C12—H12A	109.9
C1—C2—H2A	109.9	C11—C12—H12A	110.5
O2—C2—H2B	109.6	O1—C12—H12B	109.8
C1—C2—H2B	109.9	C11—C12—H12B	108.8
H2A—C2—H2B	108.3	H12A—C12—H12B	108.4
O2—C3—C4	109.6 (3)	F1—B1—F2'	102.9 (11)
O2—C3—H3A	109.9	F1—B1—F3'	133.6 (11)
C4—C3—H3A	110.5	F2'—B1—F3'	117.7 (12)
O2—C3—H3B	109.6	F1—B1—F4'	60.7 (9)
C4—C3—H3B	109.0	F2'—B1—F4'	114.0 (11)
H3A—C3—H3B	108.1	F3'—B1—F4'	115.3 (10)
O4—C6—C5	110.0 (4)	F1—B1—F3	105.8 (9)
O4—C6—H6A	109.5	F2'—B1—F3	132.3 (11)
C5—C6—H6A	110.0	F4'—B1—F3	113.2 (9)
O4—C6—H6B	109.6	F1—B1—F2	121.6 (10)
C5—C6—H6B	109.4	F3'—B1—F2	96.7 (10)
H6A—C6—H6B	108.2	F4'—B1—F2	129.6 (9)
O1—C1—C2	109.4 (3)	F3—B1—F2	113.3 (9)
O1—C1—H1D	109.9	F1—B1—F4	110.0 (8)
C2—C1—H1D	109.9	F2'—B1—F4	94.5 (9)
O1—C1—H1E	109.6	F3'—B1—F4	89.1 (9)
C2—C1—H1E	109.8	F4'—B1—F4	50.4 (8)
H1D—C1—H1E	108.3	F3—B1—F4	110.0 (8)
O4—C7—C8	109.3 (4)	F2—B1—F4	95.5 (6)
O4—C7—H7A	110.5	F1—B1—F1'	45.5 (7)
C8—C7—H7A	109.4	F2'—B1—F1'	89.1 (9)
O4—C7—H7B	109.7	F3'—B1—F1'	110.9 (11)
C8—C7—H7B	109.8	F4'—B1—F1'	106.0 (12)
H7A—C7—H7B	108.1	F3—B1—F1'	84.9 (9)
O3—C5—C6	109.2 (3)	F2—B1—F1'	96.5 (7)
O3—C5—H5A	110.7	F4—B1—F1'	155.2 (9)
C6—C5—H5A	109.9	C13—C14—C13^i^	112.0 (4)
O3—C5—H5B	108.9	C13—C14—H14A	109.6
C6—C5—H5B	110.0	C13^i^—C14—H14A	108.8
H5A—C5—H5B	108.2	N1—C13—C14	114.8 (3)
O6—C10—C9	110.3 (4)	N1—C13—H13A	108.3
O6—C10—H10A	110.0	C14—C13—H13A	108.3
C9—C10—H10A	110.4	N1—C13—H13B	108.9
O6—C10—H10B	108.9	C14—C13—H13B	108.7
C9—C10—H10B	109.0	H13A—C13—H13B	107.6
H10A—C10—H10B	108.1	F2'—F2—B1	58 (3)
O3—C4—C3	108.7 (3)	F3'—F3—B1	70.8 (17)
O3—C4—H4A	109.7	F4'—F4—B1	58.2 (6)
C3—C4—H4A	109.7	F1'—F1—B1	78.7 (9)
O3—C4—H4B	110.6	F1'—F1—F4'	140.6 (13)
C3—C4—H4B	109.7	B1—F1—F4'	62.4 (7)
H4A—C4—H4B	108.5	F1—F1'—B1	55.8 (6)
O6—C11—C12	109.5 (4)	F2—F2'—B1	101 (3)
O6—C11—H11A	110.6	F3—F3'—B1	80 (2)
C12—C11—H11A	110.4	F4—F4'—F1	126.9 (10)
O6—C11—H11B	109.4	F4—F4'—B1	71.4 (8)
C12—C11—H11B	108.6	F1—F4'—B1	57.0 (6)
H11A—C11—H11B	108.2		
			
C3—O2—C2—C1	−176.1 (3)	F4—B1—F1—F1'	−175.3 (10)
C2—O2—C3—C4	−173.6 (3)	F2'—B1—F1—F4'	110.5 (12)
C7—O4—C6—C5	−176.6 (3)	F3'—B1—F1—F4'	−98.1 (14)
C12—O1—C1—C2	−177.8 (3)	F3—B1—F1—F4'	−108.0 (10)
O2—C2—C1—O1	−65.5 (4)	F2—B1—F1—F4'	120.9 (11)
C6—O4—C7—C8	−175.9 (3)	F4—B1—F1—F4'	10.8 (9)
C4—O3—C5—C6	176.1 (4)	F1'—B1—F1—F4'	−173.9 (14)
O4—C6—C5—O3	−66.6 (4)	F4'—F1—F1'—B1	9(2)
C11—O6—C10—C9	−176.4 (4)	F2'—B1—F1'—F1	109.2 (14)
C5—O3—C4—C3	178.3 (3)	F3'—B1—F1'—F1	−131.3 (15)
O2—C3—C4—O3	66.2 (4)	F4'—B1—F1'—F1	−5.5 (13)
C10—O6—C11—C12	−172.4 (4)	F3—B1—F1'—F1	−118.2 (13)
C8—O5—C9—C10	175.9 (4)	F2—B1—F1'—F1	128.9 (12)
O6—C10—C9—O5	−65.0 (5)	F4—B1—F1'—F1	10 (2)
C9—O5—C8—C7	171.7 (3)	F1—B1—F2'—F2	155 (3)
O4—C7—C8—O5	65.7 (4)	F3'—B1—F2'—F2	−2(3)
C1—O1—C12—C11	172.4 (3)	F4'—B1—F2'—F2	−142 (3)
O6—C11—C12—O1	60.6 (5)	F3—B1—F2'—F2	29 (3)
C13^i^—C14—C13—N1	−179.2 (4)	F4—B1—F2'—F2	−94 (3)
F1—B1—F2—F2'	−29 (3)	F1'—B1—F2'—F2	111 (3)
F3'—B1—F2—F2'	178 (3)	F1—B1—F3'—F3	−20 (4)
F4'—B1—F2—F2'	47 (3)	F2'—B1—F3'—F3	128 (3)
F3—B1—F2—F2'	−157 (3)	F4'—B1—F3'—F3	−93 (3)
F4—B1—F2—F2'	88 (3)	F2—B1—F3'—F3	127 (3)
F1'—B1—F2—F2'	−70 (3)	F4—B1—F3'—F3	−137 (3)
F1—B1—F3—F3'	165 (3)	F1'—B1—F3'—F3	28 (3)
F2'—B1—F3—F3'	−70 (3)	B1—F4—F4'—F1	13.8 (11)
F4'—B1—F3—F3'	101 (3)	F1'—F1—F4'—F4	−25 (3)
F2—B1—F3—F3'	−59 (3)	B1—F1—F4'—F4	−15.7 (12)
F4—B1—F3—F3'	46 (3)	F1'—F1—F4'—B1	−9(2)
F1'—B1—F3—F3'	−154 (3)	F1—B1—F4'—F4	166.8 (11)
F1—B1—F4—F4'	−12.2 (10)	F2'—B1—F4'—F4	75.1 (12)
F2'—B1—F4—F4'	−117.7 (11)	F3'—B1—F4'—F4	−65.6 (13)
F3'—B1—F4—F4'	124.6 (11)	F3—B1—F4'—F4	−97.4 (11)
F3—B1—F4—F4'	104.0 (11)	F2—B1—F4'—F4	58.4 (14)
F2—B1—F4—F4'	−138.8 (9)	F1'—B1—F4'—F4	171.4 (9)
F1'—B1—F4—F4'	−20.1 (19)	F2'—B1—F4'—F1	−91.7 (13)
F2'—B1—F1—F1'	−75.6 (13)	F3'—B1—F4'—F1	127.6 (13)
F3'—B1—F1—F1'	76 (2)	F3—B1—F4'—F1	95.7 (11)
F4'—B1—F1—F1'	173.9 (14)	F2—B1—F4'—F1	−108.4 (14)
F3—B1—F1—F1'	65.9 (14)	F4—B1—F4'—F1	−166.8 (11)
F2—B1—F1—F1'	−65.2 (12)	F1'—B1—F4'—F1	4.5 (11)

Symmetry codes: (i) −*x*, *y*, −*z*+1/2.

### Hydrogen-bond geometry (Å, °)

**Table:**

*D*—H···*A*	*D*—H	H···*A*	*D*···*A*	*D*—H···*A*
N1—H1B···O2	0.89	2.06	2.915 (3)	161
N1—H1A···O4	0.89	2.03	2.911 (4)	169
N1—H1C···O6	0.89	2.08	2.967 (4)	179
C12—H12B···F4'	0.97	2.48	3.316 (19)	144
C13—H13A···F2	0.97	2.47	3.346 (12)	150
C13—H13A···F2'	0.97	2.42	3.361 (16)	162
C5—H5A···F3'^ii^	0.97	2.41	3.350 (18)	162
C10—H10B···F1'^iii^	0.97	2.41	3.355 (17)	166
C10—H10B···F2'^iii^	0.97	2.44	3.235 (19)	139
C8—H8A···F3'^iv^	0.97	2.50	3.412 (14)	156

Symmetry codes: (ii) −*x*, *y*, −*z*+1/2; (iii) *x*, *y*+1, *z*; (iv) −*x*, *y*+1, −*z*+1/2.
